# Binding Mode Analyses and Pharmacophore Model Development for Stilbene Derivatives as a Novel and Competitive Class of α-Glucosidase Inhibitors

**DOI:** 10.1371/journal.pone.0085827

**Published:** 2014-01-21

**Authors:** Yuno Lee, Songmi Kim, Jun Young Kim, Mahreen Arooj, Siu Kim, Swan Hwang, Byeong-Woo Kim, Ki Hun Park, Keun Woo Lee

**Affiliations:** Division of Applied Life Science (BK21 Program), Systems and Synthetic Agrobiotech Center (SSAC), Plant Molecular Biology and Biotechnology Research Center (PMBBRC), Research Institute of Natural Science (RINS), Gyeongsang National University (GNU), Jinju, Republic of Korea; UMR-S665, INSERM, Université Paris Diderot, INTS, France

## Abstract

Stilbene urea derivatives as a novel and competitive class of non-glycosidic α-glucosidase inhibitors are effective for the treatment of type II diabetes and obesity. The main purposes of our molecular modeling study are to explore the most suitable binding poses of stilbene derivatives with analyzing the binding affinity differences and finally to develop a pharmacophore model which would represents critical features responsible for α-glucosidase inhibitory activity. Three-dimensional structure of *S. cerevisiae* α-glucosidase was built by homology modeling method and the structure was used for the molecular docking study to find out the initial binding mode of compound **12**, which is the most highly active one. The initial structure was subjected to molecular dynamics (MD) simulations for protein structure adjustment at compound **12**-bound state. Based on the adjusted conformation, the more reasonable binding modes of the stilbene urea derivatives were obtained from molecular docking and MD simulations. The binding mode of the derivatives was validated by correlation analysis between experimental K_i_ value and interaction energy. Our results revealed that the binding modes of the potent inhibitors were engaged with important hydrogen bond, hydrophobic, and π-interactions. With the validated compound **12**-bound structure obtained from combining approach of docking and MD simulation, a proper four featured pharmacophore model was generated. It was also validated by comparison of fit values with the K_i_ values. Thus, these results will be helpful for understanding the relationship between binding mode and bioactivity and for designing better inhibitors from stilbene derivatives.

## Introduction

Several glucosidases catalyze the cleavage of glycosidic bonds in oligosaccharides or glycoconjugates and release glucose from the non-reducing end of the oligosaccharide chain. α-glucosidase (EC. 3.2.1.20; α-glucosidase glucohydrolase) is an enzyme that catalyzes the cleavage of glycosidic bond in maltose [1]. Inhibition of the enzyme helps to absorb less glucose and suppresses digestion of carbohydrates since the carbohydrates are not hydrolyzed to glucose molecules [2]. Moreover, glycosidase inhibitors have proven useful to reduce postprandial hyperglycemia by preventing the digestion of carbohydrates, being effective for the treatment of type II diabetes and obesity [3]-[5].

Glycosidic derivatives are potential therapeutic agents for the treatment of disorders such as human immunodeficiency virus (HIV) infection, as well as diabetes, Gaucher's disease, metastatic cancer, and lysosomal storage diseases, and can disrupt glycoprotein processing through direct-site irreversible glucosidase inhibition [6]–[8]. These derivatives have a profound role to play on this process because they mimic the disaccharide unit which is cleaved by glucosidases [9]. Most of the glucosidase inhibitors are glycosidic derivatives and there are only few non-glycosidic derivatives which effectively inhibit glucosidases [10]. Recently our report on non-glycosidic derivatives demonstrated that readily accessible achiral (*E*)-1-phenyl-3-(4-strylphenyl)urea derivatives are potent competitive α-glucosidase inhibitors with very micromolar IC_50_s [11].

**Figure 1:**
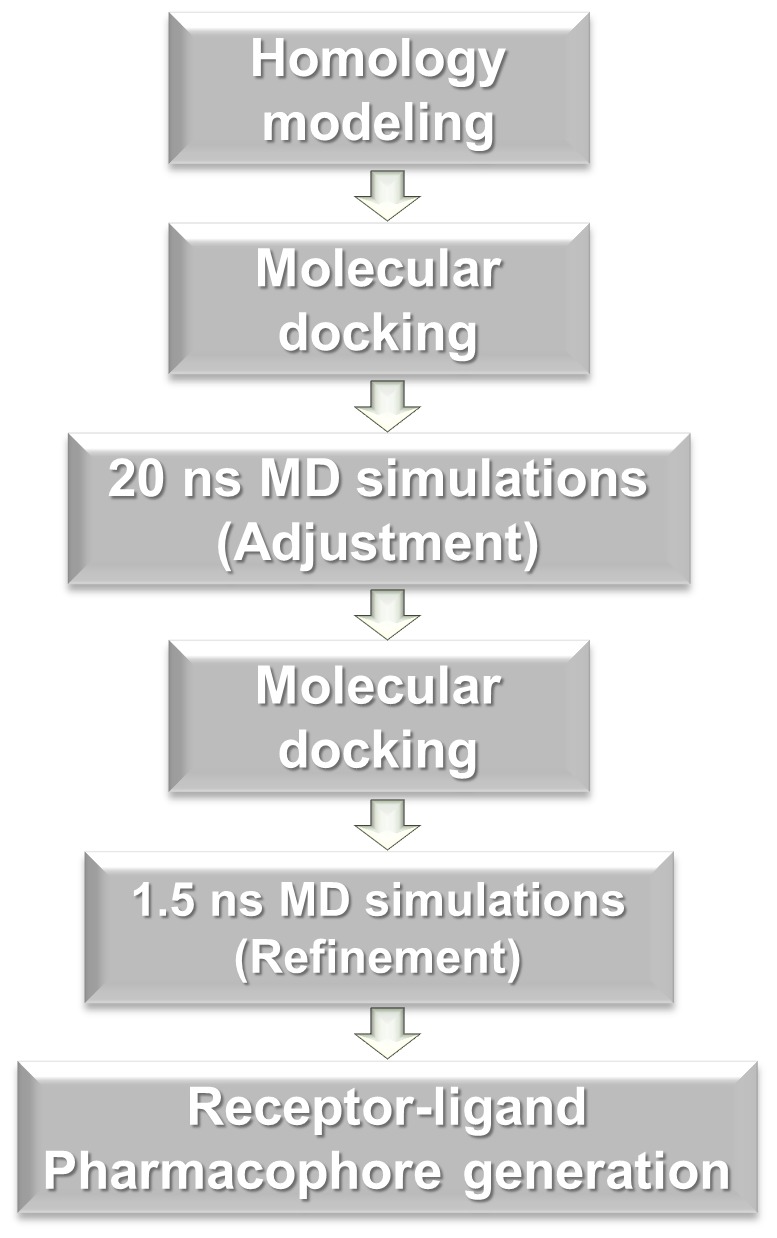
Workflow of combining molecular docking and molecular dynamics simulation approaches for indentifying the reasonable binding site and generating the proper pharmacophore model.

The main purposes of the present study are to find out the reasonable binding mode between the stilbene derivative and the protein and to generate pharmacophore model using the protein-ligand complex structure. To identify the reasonable binding mode, homology modeled structure of *S. cerevisiae* α-glucosidase is used for molecular modeling study. However, in general, finding the binding mode for an induced-fit model such as α-glucosidase is very difficult because it has many loops in active site. Thus, here, new approach was introduced to solve this problem. Combined molecular modeling studies including molecular docking and molecular dynamics (MD) simulations were carried out to investigate structural rationales for the inhibitory activities of the stilbene derivatives, especially for compounds **6** and **12** (Figure 1). The compound **12** has two fluorine atoms on the C ring, while compound **6** has hydrogen atoms instead of fluorine. This subtle structural difference of the **12** with **6** makes much difference in binding affinities. Hence, to find out the proper reason for this, the MD simulations were performed two times for two different purposes: i) for adjustment of protein structure with the most active molecule, compound **12**, and ii) for refinement of final docking poses. Based on these results, finally we have developed a reasonable pharmacophore model using receptor-ligand pharmacophore generation method.

## Results/Discussion

### Structure generation and validation of *S. cerevisiae* α-glucosidase

**Figure 2:**
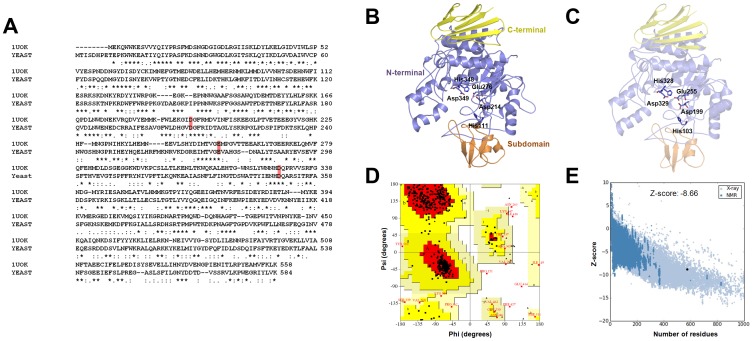
Sequence alignment and homology modeling for *S. cerevisiae* α-glucosidase using a template *B. cereus* oligo-1,6-glucosidase. (A) Sequence alignment of *S. cerevisiae* α-glucosidase (represented as YEAST) with *B. cereus* oligo-1,6-glucosidase (1UOK). Sequence identities are denoted by asterisks (*), conservative substitutions by colons (:), and semi-conservative substitutions by dots (.). The catalytic residues are indicated in a red box. Comparative view of the homology modeled structure *S. cerevisiae* α-glucosidase (B), the template structure of *B. cereus* oligo-1,6-glucosidase (PDB ID: 1UOK) (C) with the conserved catalytic residues represented as sticks. The N-terminal, subdomain, and C-terminal domains are shown in blue, orange, and yellow, respectively. (D) Ramachandran plot of the *φ*/*ψ* distribution of the homology model as obtained by PROCHECK. (E) Z-score plot for our modeled structure shows that the score is within the range of scores typically found for native proteins of similar size.

The α-glucosidase from *S. cerevisiae* was used in biological testing of the inhibitors for present study. The 3D structure of the protein is required to investigate the binding mode of stilbene derivatives within the α-glucosidase structure. The homology modeling of the protein has already been reported in several publications [12]–[14]. To construct the 3D structure of the α-glucosidase, homology modeling method was used like that of the previous studies. The structure of oligo-1,6-glucosidase from *B. cereus* (PDB ID: 1UOK) [15] was selected as template and the sequence alignment between α-glucosidase and the template was carried out using ClustalW2 package [16] (Figure 2A). According to this alignment, the α-glucosidase shares around 38.0% sequence identity and 62.0% sequence similarity with the template.

The 3D structure of α-glucosidase was generated by *Build homology models protocol* which implements MODELER program available in Discovery Studio (DS) 3.0 software [17]. The homology model was built by omitting the first 8 residues at the N-terminal region, since no sequence similarity was found for N-terminal residues from the sequence alignment. Figure 2B and 2C show the modeled structure of α-glucosidase compared with X-ray crystal structure of the template representing the three domains: the N-terminal, the subdomain, and the C-terminal domain. The catalytic triad residues (Asp199, Glu255, and Asp329) are found in the N-terminal domain of the template protein [15] while the catalytic triad in the α-glucosidase is formed by Asp214, Glu276 and Asp349 residues, respectively. The substrate binding site is located in the cleft between the N-terminal domain and the subdomain. Two His residues near to center of catalytic triad, His103 and His328 of oligo-1,6-glucosidase which may be required for substrate binding are also conserved in α-glucosidase (His111 and His348, respectively) [18].

The final structure of α-glucosidase generated from homology modeling was evaluated by two programs namely PROCHECK [19] and ProSA [20] to check the stereochemical quality. The ramachandran plot obtained by PROCHECK program showed that 87.5% of residues of the final 3D structure lied in most favored regions better than that of the X-ray crystal structure of the template which has 86.3% residues (Figure 2D). The ProSA z-score value of the final model structure is −8.66 and the plot indicates that the overall model quality is within the range of scores typically found for proteins of similar size (Figure 2E).

### Initial molecular docking results and validation

**Figure 3:**
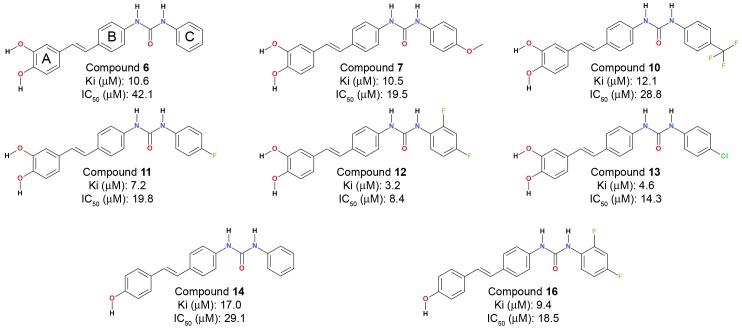
2D chemical structures of stilbene derivatives with experimental binding affinity value such as K_i_ and IC_50_.

In order to gain insight into the most probable binding modes of the stilbene derivatives within the active site of α-glucosidase, the molecular docking simulations of the reported derivatives ([11], Figure 3) were performed with the modeled structure of α-glucosidase using CDOCKER program [21].

**Figure 4:**
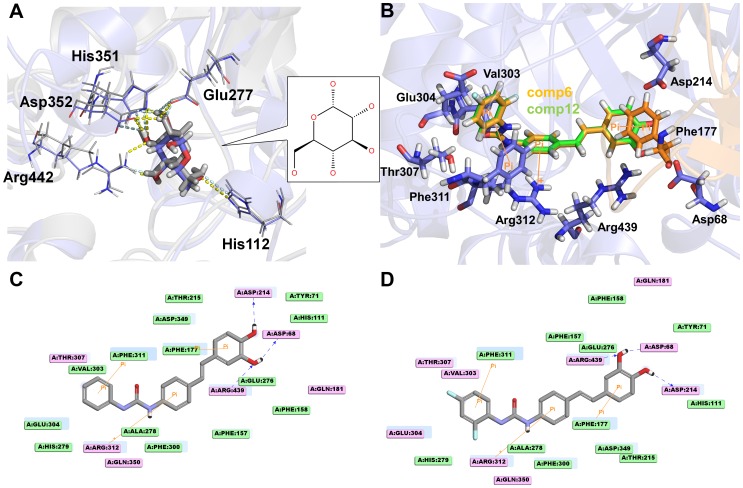
Results of initial molecular docking simulation. (A) Validation of molecular docking simulation by comparison between crystal structure (gray) of *S. cerevisiae* isomaltase (PDB ID: 3A4A) co-crystallized with the α-D-glucose and homology modeled structure (blue) of the isomaltase with its docking pose. Hydrogen bonds are represented as dotted lines in crystal (cyan) and homology modeled (yellow) structures. 2D structure of α-D-glucose is shown in the right box. (B) Initial molecular docking results of compound **6** (orange) and compound **12** (green) with representing interacting residues which shown as sticks. 2D interaction diagram of compound **6** (C) and compound **12** (D) with representing charged (pink plate), π (orange line), and hydrophobic (light green plate) interacting residues.

For validating the CDOCKER docking protocol, the crystal structure of isomaltase from *S. cerevisiae* (PDB ID: 3A4A) co-crystallized with the α-D-glucose, which is part of inhibitor maltose, was used for additional homology modeling and docking simulation. Although the 2D structure of maltose is not similar with the further docked compounds, the sequence identity (39.6%) of this isomaltase enzyme with the template from *B. cereus* is similar to that (38%) of the modeled enzyme with the template (Figure S1A). Hence, we selected this different enzyme for validation process. To reproduce same protocol, homology modeling of the isomaltase was carried out using the template from *B. cereus* and then the modeled isomaltase was compared with its own crystal structure (Figure S1B). The root mean square deviation (RMSD) value between the homology model and crystal structure of isomaltase is 0.22 nm indicating that the homology model of α-glucosidase built by the template was validated. Subsequently, the docking simulation was performed based on the modeled isomaltase with the α-D-glucose. We compared the crystallographic conformation & position of α-D-glucose in the X-ray structure of the complex to its poses obtained by docking (Figure 4A). The hydrogen bond interactions of α-D-glucose with active site residues His112, Glu277, His351, Asp352, and Arg442 in the crystal structure also appeared in the docked poses of α-D-glucose. The root mean square deviation (RMSD) between the crystal and docked structure is 0.11 nm. This validation has proven that our docking protocol was reasonable in investigating the binding conformation accurately.

All the derivatives were well docked into the active site of the modeled structure. Due to the same scaffold of the derivatives, binding modes of the derivatives were almost same each other. From the binding mode comparison between compounds **6** and **12**, same hydrogen bond, π, and hydrophobic interactions were observed with only few different interactions (Figures 4B to 4D). Moreover, the negative CDOCKER energy score (34.31) of compound **6** is similar to the score (36.1) of compound **12**. These results indicate that the docking simulation is not enough to explain the activity difference between compounds **6** and **12**. Thus, molecular dynamics (MD) simulation was implemented to better understand this difference. To do this, an improved binding mode of the derivatives was required. Hence the initial docking pose of compound **12** which is the most active one was subjected into MD simulation during 20 ns.

### Selection of the best adjusted structure using interaction energy and negative CDOCKER energy

**Figure 5:**
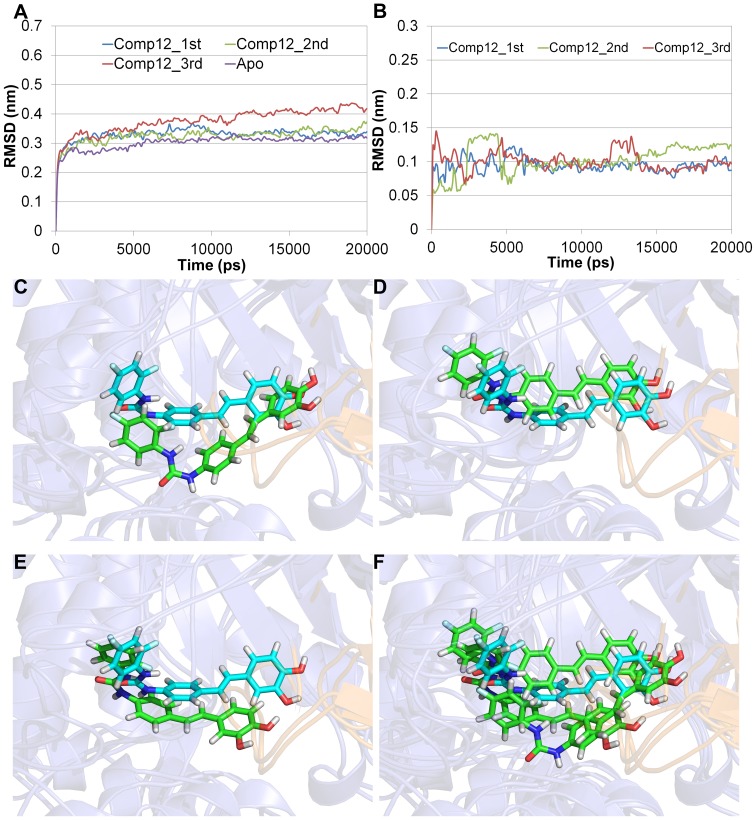
MD simulation results of four systems (Apo, first, second, and third trials) for structure adjustment of compound 12-bound state. Cα RMSD plot of three trials and Apo systems (A), RMSD of ligand in three trial systems (B). First trial (C), second (D), third (E), and all three representative structures (F) with initial docking pose colored by cyan.

Four 20 ns MD simulations (Apo, first, second, and third trials) were carried out to obtain a reasonable structure adjusted in compound **12**-bound state. These three trial simulations are started from the same system with same conditions to approach global minimum conformation of the complex. The Cα RMSD analysis showed that all systems were well stabilized at around 0.3 nm (Figure 5A). Although the third trial system has relatively higher Cα RMSD than the other systems, the RMSD of compound **12** is well maintained after 15 ns. The value of RMSD for compound **12** is measured, after superimposition of ligand conformations in initial and each time step. In first and second trial systems, compound **12** is also well stabilized (Figure 5B). Based on the RMSD results, the closest frame to average structure during the last 5 ns was selected as the representative structure of each trial. From the comparison of initial docked structure and the three representative structures, similar binding mode was observed but average poses mostly different from each other (Figure 5C to 5F). Among these different three local minima, global minimum conformation, the best adjusted conformation for compound **12**, was selected by computing and comparing averaged interaction energies (sum of columbic and van der Waals energies) in the last 5 ns of simulation (Table 1). Some publications reported that correlation of the interaction energy with binding affinity can be observed positively [22], [23]. The lowest averaged interaction energy (−253.253 kJ/mol) was shown in the second trial system compared to the other systems. Hence, we concluded that the adjusted protein structure in the second trial system is the closest to the global minimum conformation.

**Table 1:** Averaged interaction energy of compound **12** obtained from MD simulation.

System	Interaction Energy	Van der Waals Energy	Electrostatic Energy
	(kJ/mol)	(kJ/mol)	(kJ/mol)
Comp12_1st	−235.876	−168.048	−67.8278
Comp12_2nd	−253.253	−202.956	−50.2964
Comp12_3rd	−203.842	−159.364	−44.4776

**Figure 6:**
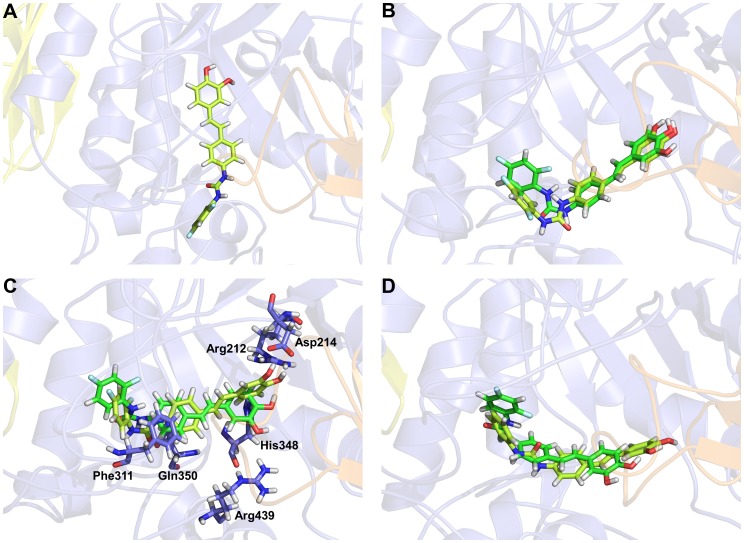
Molecular docking results of compound 12. The best docking poses (light green) of compound 12 in adjusted protein structure of Apo (A), first (B), second (C), third (D) trial systems with comparing the MD results which are represented by dark green.

In order to check whether the lowest energy structure is more reasonable for binding of stilbene derivatives than the homology modeled one, negative CDOCKER energies were also compared after conducting a several molecular docking simulations of compound **12** with the representative protein conformations (Figure 6). As expected, the lowest interaction energy value of −73.0613 was observed in the docked pose of compound **12** in second trial system (Table 2). The negative CDOCKER energy score of the second trial system was also lower than the initial docking structure as well as the other Apo and trial systems. Although the second lowest energy score (−66.9361) was detected in the docked pose of compound **12** in Apo system, binding pose of compound **12** was not proper because many flexible loops in active site were gathered and then the possible ligand binding cavity was removed (Figure 6A).

**Table 2:** Interaction energy and negative CDOCKER energy of compound **12** obtained from molecular docking simulation.

System	Interaction Energy	Van der Waals Energy	Electrostatic Energy	−CDOCKER Energy	−CDOCKER Interaction Energy
	(kJ/mol)	(kJ/mol)	(kJ/mol)		
Initial (CDOCKER)	−59.2922	−41.7959	−17.4963	36.098	47.5829
Apo	−66.9361	−43.52	−23.4162	39.1709	51.3061
Comp12_1st	−59.0961	−43.039	−16.0571	35.7489	49.0586
Comp12_2nd	−73.0613	−48.1349	−24.9264	44.1057	57.4398
Comp12_3rd	−57.3609	−38.5771	−18.7839	31.1857	44.715

**Figure 7:**
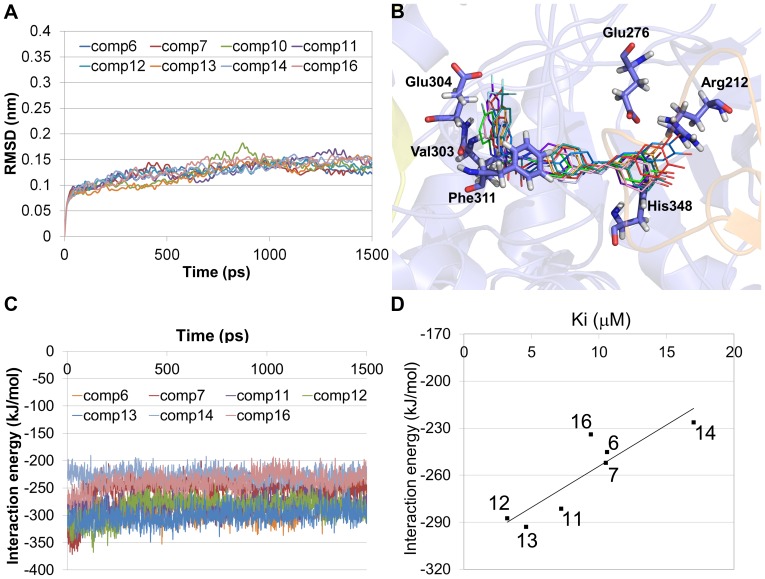
Results of MD simulations of stilbene derivatives starting with docked structure in the second trial system. (A) Cα RMSD plot of different stilbene derivative-bound systems. (B) Overlapped structure of compound **6** (orange), compound **7** (red), compound **10** (bluish green), compound **11** (violet), compound **12** (green), compound **13** (sky blue), compound **14** (light blue), and compound **16** (light violet). (C) Interaction energy plot of all systems during the 1.5 ns simulation time. (D) Correlation graph between experimental K_i_ value and interaction energy.

Three trial MD simulations of initial docked compound **6**-bound system were also conducted to check whether the homology modeled structure which is in Apo state is reasonable to bind the derivatives. As clearly shown in compound **6**-bound trial systems as well as compound **12**-bound trial systems, the results of MD simulation with compound **6** were deviated from the proper binding region indicating that inappropriate starting conformation of the protein was used. But, after using the best adjusted conformation in the compound **12**-bound state, the structure of compound **6** was maintained stably during the simulation time (Figure 7A). From these results, we can suppose that the best adjusted conformation by the most active compound was required to find a more reasonable binding mode of the derivatives at least in this system.

These comparative analyses suggested that the adjusted protein conformation in second trial system is the suitable structure rather than the other ones. Thus, the lowest energy protein structure was used for molecular docking simulation of the derivatives.

### Molecular docking and molecular dynamics simulations with the adjusted protein conformation

In order to find out the most reasonable binding mode, molecular docking and MD simulations of the derivatives were performed with the adjusted protein structure having lowest energy conformation. Initial docking poses of derivatives in adjusted protein structure were well overlaid in same binding mode showing only subtle difference in the C rings (Figure S3). These poses have lower scores than the first docking results. But, to obtain more refined poses, MD simulations were conducted. Hence eight 1.5 ns MD simulations were carried out and analyzed (Figure 7). Unlike the simulation results of the homology modeled structure in Apo state, all the structures are well converged in similar binding mode (Figure 7B). The Cα RMSDs of the systems also showed that the deviation (around 0.15 nm) of the structure from initial one was much lower than previous MD simulations (around 0.30 nm) at the same time (Figure 7A). In addition, calculated interaction energies were maintained stably for almost compounds (Figure 7C). But, the electrostatic energy of compound **10** was highly unstable compared to the other system (Figure S2). These results indicate that the structures except for compound **10**-bound state are maintained stably during the simulation time due to the adjusted protein conformation.

In order to evaluate whether the binding mode is reasonable, correlation was calculated between experimental K_i_ value and the interaction energy obtained from the eight 1.5 ns MD simulations. As a result, the correlation coefficient value was 0.89 (Figure 7D). This means that there's a positive linear correlation between calculated interaction energy and experimental K_i_ value (Table 3). Thus, the binding mode of the derivatives with the protein is suitable to use receptor-ligand pharmacophore model generation.

**Table 3:** Correlation between exp. K_i_ and calculated energy.

System	Experimental K_i_	Interaction Energy
	(µM)	(kJ/mol)
Comp6	10.6	−245.265
Comp7	10.5	−252.126
Comp11	7.2	−281.353
Comp12	3.2	−287.378
Comp13	4.6	−292.982
Comp14	17	−226.249
Comp16	9.4	−233.907

### Comparison of final docking poses refined by MD simulation

When the final binding modes of the derivatives were compared, those of the two compounds **6** and **12** on behalf of eight derivatives were focused on to analyze the differences of their binding affinities because the compound **12** is the most active molecule and the structural difference of the **12** with **6** makes much difference in binding affinities. The structural difference is that compound **12** has two fluorine atoms on the C ring, while compound **6** has hydrogen atoms instead of fluorine. Due to this subtle difference, the binding affinity of **12** is 3 folds higher than of **6**. To analyze the reasons for this, the binding modes of the compounds were compared using the 1.5 ns snapshot. Several differences were observed in hydrogen bonding interaction but the other interactions are similar with each other. Hence, to find out the clear reason of the activity difference and to obtain more refined binding mode of the compounds, two MD simulations of compounds **6** and **12** bound systems were extended to 10 ns. To quantitatively compare binding mode difference between 1.5 ns and 10 ns simulations, final snapshot of 1.5 ns and representative structure (9,046 ps) of 10 ns simulations were superimposed by protein and then RMSD of protein (0.15 nm) and compound **12** (0.09 nm) was calculated and compared between two simulations. From the result, we found that the binding modes obtained from the two simulations are similar with each other. But, we wanted to obtain more refined structure for further analysis and pharmacophore model generation. Hence, the closest frame to average structure during the last 2 ns was selected as representative structure.

**Figure 8:**
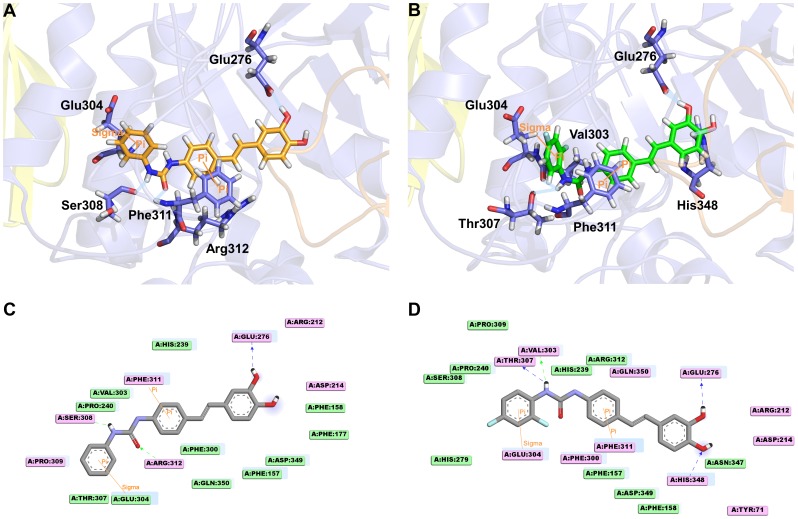
Binding modes of compound 6 (orange) and compound 12 (green) obtained from 10 ns MD simulation. (A) Hydrogen bonding interactions (light blue line) of compound **6** with Glu276, Ser308, and Arg312 are displayed with π-interacting residues (orange line). (B) Interactions of compound **12** with four hydrogen bonding residues including Glu276, Val303, Thr307, and His348 are represented with π-interacting residues: Glu304 for π-sigma and Phe311 for π-π interactions. 2D interaction diagram of compound **6** (C) and compound **12** (D) with representing charged (pink plate), π (orange line), and hydrophobic (light blue plate) interacting residues.

Many different interacting points are observed upon comparison of the refined docking conformations for **6** and **12** by analyzing through monitor command in DS 3.0. Whereas the compound **12** was found to have four hydrogen bonding interactions with Glu276, Val303, Thr307, and His348, the compound **6** having no fluorine atom has formed three hydrogen bonds with Glu276, Ser308, and Arg312 (Table 4). The time occupancies of hydrogen bonds for compound **6** are relatively higher than those for compound **12**. But, one of the hydroxyl groups of the A ring in both compounds formed strong hydrogen bond interaction with Glu276 which is one of catalytic triad residues (Figure 8). The number of interacting residues involved in charge or polar interaction in **12** was higher than in **6**. In π interactions comparison, same π-sigma and π-π interactions in both compounds were formed with Glu304 and Phe311, respectively.

**Table 4:** Hydrogen bonding and hydrophobic contacting residues between protein and compound.

Ligand	Protein-ligand interactions		
	Hydrogen bonding residues (Time occupancy during the last 2 ns)	Residues involved in charge or polar interaction	Hydrophobic contacting residues
Compound 6	**Glu276 (99.95%)**, Ser308 (80.95%), Arg312 (97.65%)	**Arg212**, **Asp214**, Pro309, **Phe311**	**Phe157**, **Phe158**, Phe177, **His239**, **Pro240**, Phe300, Val303, Glu304, Thr307, **Asp349**, Gln350
Compound 12	**Glu276 (99.75%)**, Val303 (26.6%), Thr307 (67.2%), His348 (57.35%)	Tyr71, **Arg212**, **Asp214**, Glu304, Phe300, **Phe311**, Gln350	**Phe157**, **Phe158**, **His239**, **Pro240**, His279, Ser308, Pro309, Arg312, Asn347, **Asp349**

**Figure 9:**
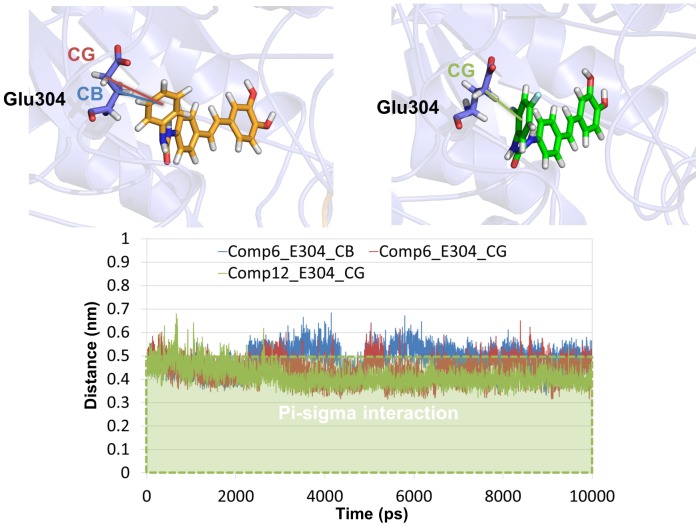
Distance of π-sigma interaction between Glu304 and each compound. Distance of compound **12** with β-carbon and γ-carbon of Glu304 is represented by blue and red lines, respectively (upper left). Distance between γ-carbon of Glu304 and compound **12** is shown as green line (upper right). These three distance values are compared during the simulation time (bottom). The threshold of π-sigma interaction is highlighted by green dotted box.

In order to provide another clear reason of the activity difference in terms of dynamic behavior, distances of π-sigma interaction between Glu304 and each compound were measured and compared during the simulation time (Figure 9). The distance should be less than 0.5 nm to form a π-sigma interaction [24]. Some cases showed that involvement of π-sigma interaction play an important role in protein-ligand interaction [25]–[27]. Whereas the distance between γ-carbon of Glu304 and compound **12** was maintained stable, the distance in case of compound **6** was deviated out of the range. This indicated that the π-sigma interaction could also be one of the key interactions to explain the activity difference in terms of dynamic behavior.

To find out the effect of π -sigma interaction, interaction energy difference of the several snapshots with and without π-sigma interaction was calculated. To compare mostly similar frames excepting the π-sigma interaction only, several 1 ps different snapshots were selected and then differences of interaction energy between each two snapshots were calculated. For example, the difference between 9,784 ps (−307.62) and 9,785 ps (−280.93) snapshots was 26.69 kJ/mol and each distance of π-sigma interaction between Glu304 β or γ-carbons and compound **6** is 0.47 or 0.36 nm for 9,784 ps and 0.56 or 0.54 nm for 9,785 ps snapshot, respectively. The difference of van der Waals energies between 9,784 ps (−189.51) and 9,785 ps (−165.43) snapshots was more significantly involved than that of the electrostatic energies. In this way, we have selected 19 snapshot pairs and listed all the distance and energy difference values for these snapshots (Table S1) and then average value was calculated for these energy differences. Through these comparison analyses, we can estimate that the range of energy for a π-sigma interaction might be around −13.74±11.26 kJ/mol in our system. In conclusion of all these interaction results, we suggest that the activity difference can be explained by considering not only hydrogen and charge interactions but also π-sigma interaction.

### Receptor-ligand pharmacophore model generation

**Figure 10:**
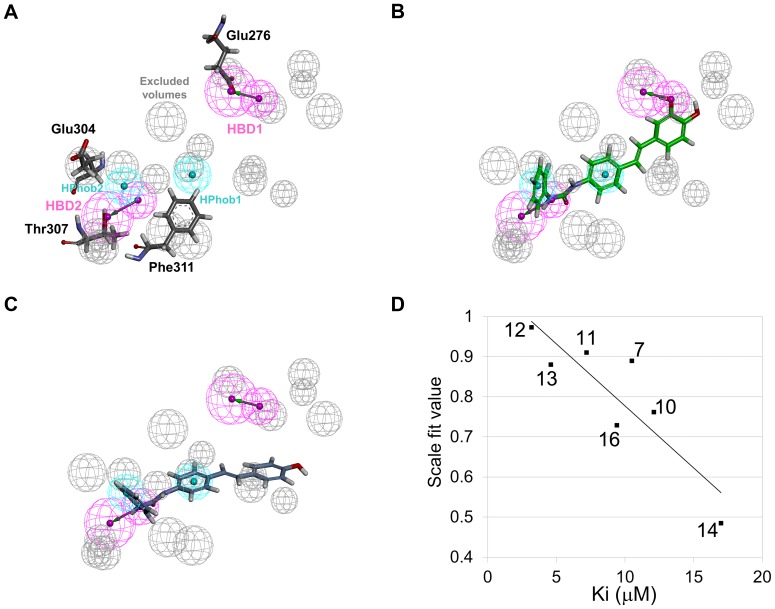
Receptor-ligand pharmacophore model generation and validation. (A) Four featured pharmacophore model consists of two hydrogen bond donors (HBD), two hydrophobic (HPhob), and excluded volumes. Mapping of generated pharmacophore model on compound **12** (B) and compound **14** (C). (D) Correlation graph between experimental K_i_ and scale fit values.

Finally the receptor-ligand pharmacophore model was generated based on the representative structure of compound **12**-bound system which is the closest frame to average structure during the last 2 ns (Figure 10A). All the four features namely two hydrogen bond donors (HBD) and two hydrophobic (HPhob) were mapped onto eight derivative compounds. Mapping of generated pharmacophore model on compound **12** and compound **14**, the most active and least ones, is shown in Figure 10B and 10C, respectively. The compound **12** taken as reference mapped well with all features with a scale fit value 0.97. The values of the other compounds are also in a good agreement with the experimental K_i_ values (Table 5). But the compound **6** shows relatively higher fit value than estimated one, because it has same conformation with compound **12**. In our pharmacophore model, π-sigma interaction between the compound and Glu304 was represented as hydrophobic feature (HPhob2). Thus, the MD simulation is required to compare these two compounds and to explain this interaction. As validation of pharmacophore model, the correlation between the scale fit value and the K_i_ value was calculated except for this specific case. The correlation coefficient was 0.88 meaning that the two different kinds of values are in positive linear correlation (Figure 10D).

**Table 5:** Correlation between exp. K_i_ and scale fit value.

System	Time, ns	Exp. K_i_ (µM)	Scale fit value
Comp6	10 (1.5)	10.6	0.93 (0.95)^a^
Comp7	1.5	10.5	0.89
Comp10	1.5	12.1	0.76
Comp11	1.5	7.2	0.91
Comp12	10 (1.5)	3.2	0.97 (0.95)^a^
Comp13	1.5	4.6	0.88
Comp14	1.5	17	0.49
Comp16	1.5	9.4	0.73

a Scale fit value of each conformation for compounds **6** and **12** after 1.5 ns.

In order to check the conservation of the residues implicated in pharmacophore between the modeled yeast enzyme and the corresponding human enzyme, sequence alignment of *S. cerevisiae* α-glucosidase with human α-amylase was performed. Although the overall sequence identity (17.4%) and similarity (34.1%) of modeled yeast and human enzymes are in low level, sequence identity (33.3%) and similarity (52.4%) of interaction residues for compound **12** is much higher than those of the rest sequences (Figure S4). The catalytic triad residues Asp214, Glu276, and Asp349 and substrate binding residues His111 and His348 are identical between human and yeast. These results suggested that our pharmacophore model is also useful for human α-amylase which is the main pharmaceutical target for stibene derivatives.

## Conclusions

The main purposes of this study were to find out the most suitable binding conformations of stilbene derivatives and to explain the differences of binding affinity and then finally to develop a receptor-ligand pharmacophore model. We constructed the homology modeled structure of *S. cerevisiae* α-glucosidase referenced by published information and used it for the molecular docking study to find out the initial binding mode of compound **12** which is the most active one. For proper protein structure adjustment at compound **12**-bound state, three 20 ns molecular dynamics (MD) simulations of the initial complex structure were performed. The representative structure of second trial system was selected as the best adjusted structure by comparing the interaction energies and negative CDOCKER energies. Based on the adjusted conformation, the most reasonable binding modes of the stilbene urea derivatives were obtained from molecular docking and MD simulations. To validate the binding mode of the derivatives, correlation analysis was conducted between experimental K_i_ value and the obtained interaction energy. From this analysis, positive linear correlation was observed with correlation coefficient value of 0.89. Our interaction analyses revealed that the binding modes of the potent inhibitors were engaged with important hydrogen bond, hydrophobic, and π-interactions. Especially, π-sigma interaction of Glu304 with each compound could also be one of the key reasons to explain the activity difference in terms of dynamic behavior. Finally, a proper four featured pharmacophore model was generated using the validated compound **12**-bound structure obtained from combining approach of docking and MD simulation. Interestingly, we also obtained a good agreement between the experimental K_i_ and the calculated fit values. These results will be helpful for understanding the relationship between binding mode and bioactivity of the stilbene derivatives and then for designing better inhibitor.

## Methods

### Homology modeling

The 3D structure of *S.cerevisiae* α-glucosidase was built by homology modeling method. The crystal structure of *B. cereus* oligo-1,6-glucosidase (PDB ID: 1UOK, 2.00 Å resolution) was used as template. Sequence alignment between *S.cerevisiae* α-glucosidase and the template was carried out using ClustalW2 package in EMBL-EBI (www.ebi.ac.uk). The Build homology models protocol available in Discovery Studio (DS) 3.0 software [17] was used to create 3D structure of *S.cerevisiae* α-glucosidase sequence based on an alignment with template. The final structure was validated by PROCHECK [19] for the evaluation of ramachandran plot and Protein Structure Analysis (ProSA) [20] from ProSA-web.

### Molecular docking simulation

For molecular modeling study, we mainly used two different programs such as CDOCKER and GROMACS for the respective purpose, i) to generate a docking pose and ii) to refine the pose within a solvated system. CHARMm force field in CDOCKER program is only used in active site region, but the Amber force field in GROMACS is used for whole system including protein, ligand, water, and ions. The CDOCKER [21] which has a significant advantage in full ligand flexibility including bonds, angles, and dihedrals is a CHARMm based docking tool to predict putative geometry of a protein-ligand complex. The CDOCKER docking simulations were performed to evaluate the binding mode of stilbene derivatives within active site of homology modeled α-glucosidase. The centroid point was generated at the center of the catalytic triad which consists of Asp214, Glu276, and Asp349 in the protein and the active site defined as 15 Å around it. Hundred ligand conformations were generated from each initial ligand structure through high temperature (1,000 K) MD simulation (1,000 steps), followed by random rotations. The conformations were then translated into the defined active site. Then candidate poses were created by dynamics based simulated annealing refinement. In the refinement, the temperature is heated up to 700 K for 2,000 steps and then cooled to 300K for 5,000 steps. Out of top 20 docked poses, a docking pose with the highest negative CDOCKER energy was only used for comparison. The interaction energy (including van der Waals and electrostatics) was calculated after docking process. The docking methodology was validated with co-crystallized α-D-glucose, part of maltose, which is competitive inhibitor of the *Saccharomyces cerevisiae* isomaltase (PDB ID: 3A4A) by comparing the initial binding conformation in crystal structure and docked pose obtained from docking simulation of the α-D-glucose into the homology model of isomaltase structure. Docking modes and binding interactions were analyzed by 2D diagram visualization and monitor command in DS 3.0 software [17].

### Molecular dynamics simulation

Totally, 12 MD simulations were performed using the GROMACS program (version 4.5.3) [28], [29] with AMBER03 [30] force field. The initial structure was immersed in an orthorhombic water box (1 nm thickness) and the net charge was neutralized by the addition of NA^+^ counterions. The long range electrostatic interactions were calculated by the particle mesh Ewald (PME) method [31]. In entire system, protein alone consists of 9,293 atoms and is made up of approximately 80,000 atoms which include about 23,000 water molecules. The general Amber force field (GAFF) [32] parameter was used for the compounds and the atomic partial charges were calculated by the semi-empirical quantum chemistry program SQM [33] via ANTECHAMBER 1.5 [34] and ACPYPE web portal (http://www.ccpn.ac.uk/ccpn/software/acpype/). The systems were subjected to a steepest descent energy minimization process to remove possible bad contacts from initial structures until a tolerance of 1,000 kJ/mol. During the system equilibration process the heavy atoms were restrained and the solvent molecules with the counterions were allowed to move during the 100 ps under NPT conditions at 300 K. Bonds between heavy atoms and corresponding hydrogen atoms were constrained to their equilibrium bond lengths using the LINCS algorithm [35]. The equilibrated structures were used to perform the production runs. A constant temperature and pressure for the whole system (300 K and 1 bar) are achieved with the V-rescale thermostat [36] and Parrinello-Rahman [37] barostat. The time step for the simulations was set to 2 fs and the coordinate data were written to the file every pico second (ps). All the analyses of the MD simulations were carried out by GROMACS, DS 3.0, and VMD software. To analyze the protein-compound interactions for the final MD simulation result, monitor command in DS3.0 was used. Each threshold for hydrogen bond distance and D-H-A angle range is set to about 3.0 Å and from about 90 to 180 degrees, respectively. VMD analysis tool [38] was also used to calculate the hydrogen bond occupancy (%) with same distance and angle range thresholds used in DS3.0.

### Pharmacophore generation and validation

The representative structure taken from final 10 ns MD simulation of compound **12**-bound system was used to generate the receptor-ligand pharmacophore model finding the pharmacophoric features in the active site and important for ligand binding. Four to six features (default) in the receptor-ligand pharmacophore generation algorithm were chosen to extract useful pharmacophores of reasonable size from all the receptor-ligand interactions. Receptor-ligand pharmacophore generation was carried out by pharmacophore generation tools in DS 3.0 software with default parameters for further use in the screening for new lead derivatives. This protocol generates selective pharmacophore models from the features corresponding to the receptor-ligand interactions. Based on the generated pharmacophore model, scale fit values of the stilbene urea derivatives were calculated by ligand pharmacophore mapping tools implemented in DS. For calculating the fit value, conformation of each compound in the final snapshot of 1.5 ns MD simulation was used. Fitting method was set to flexible which is slightly modified to better fit into the pharmacophore model. As validation of the generated pharmacophore model, the correlation analysis was conducted between the scale fit value and the K_i_ value.

## Supporting Information

Figure S1 **Sequence alignment and homology modeling structure of** ***S. cerevisiae*** **isomaltase using a template** ***B. cereus*** **oligo-1,6-glucosidase.** (A) Sequence alignment of *S. cerevisiae* isomaltase (represented as 3A4A) with oligo-1,6-glucosidase (1UOK). The catalytic residues are indicated in a red box. (B) Comparative view of the homology modeled structure of *S. cerevisiae* isomaltase constructed by the template with its own crystal structure (PDB ID: 3A4A). The conserved catalytic residues represented as sticks. The N-terminal, subdomain, and C-terminal domains for the homology model are shown in blue, orange, and yellow, respectively. The crystal structure of isomaltase is colored by black.
(TIF)

Figure S2 **Electrostatic energy plot of all systems during the 1.5 ns simulation time.** Energy values for all the other compounds are represented as transparent colors to highlight the energy values for compound **10**.
(TIF)

Figure S3 **Docking poses of stilbene derivatives in adjusted protein structure of the second trial system, which is the lowest energy conformation, with interacting residues which are highlighted by violet sticks.**
(TIF)

Figure S4 **Sequence alignment of** ***S. cerevisiae*** **α-glucosidase (represented as YEAST) with human α-amylase (Human).** Each identical, conserved, and non-conserved interacting residue is indicated in a red, yellow, and black box, respectively. Sequence identities are denoted by asterisks (*), conservative substitutions by colons (:), and semi-conservative substitutions by dots (.).
(TIF)

Table S1 **Interaction energy difference obtained by comparing mostly similar frames excepting π-sigma interaction.**
(DOCX)
