# Supplementary figures and images for: Binding Mode Analyses and Pharmacophore Model Development for Stilbene Derivatives as a Novel and Competitive Class of α-Glucosidase Inhibitors
Source: PLoS One. 2014 Jan 21;9(1):e85827. doi: 10.1371/journal.pone.0085827 (PMC3897524; doi:10.1371/journal.pone.0085827)

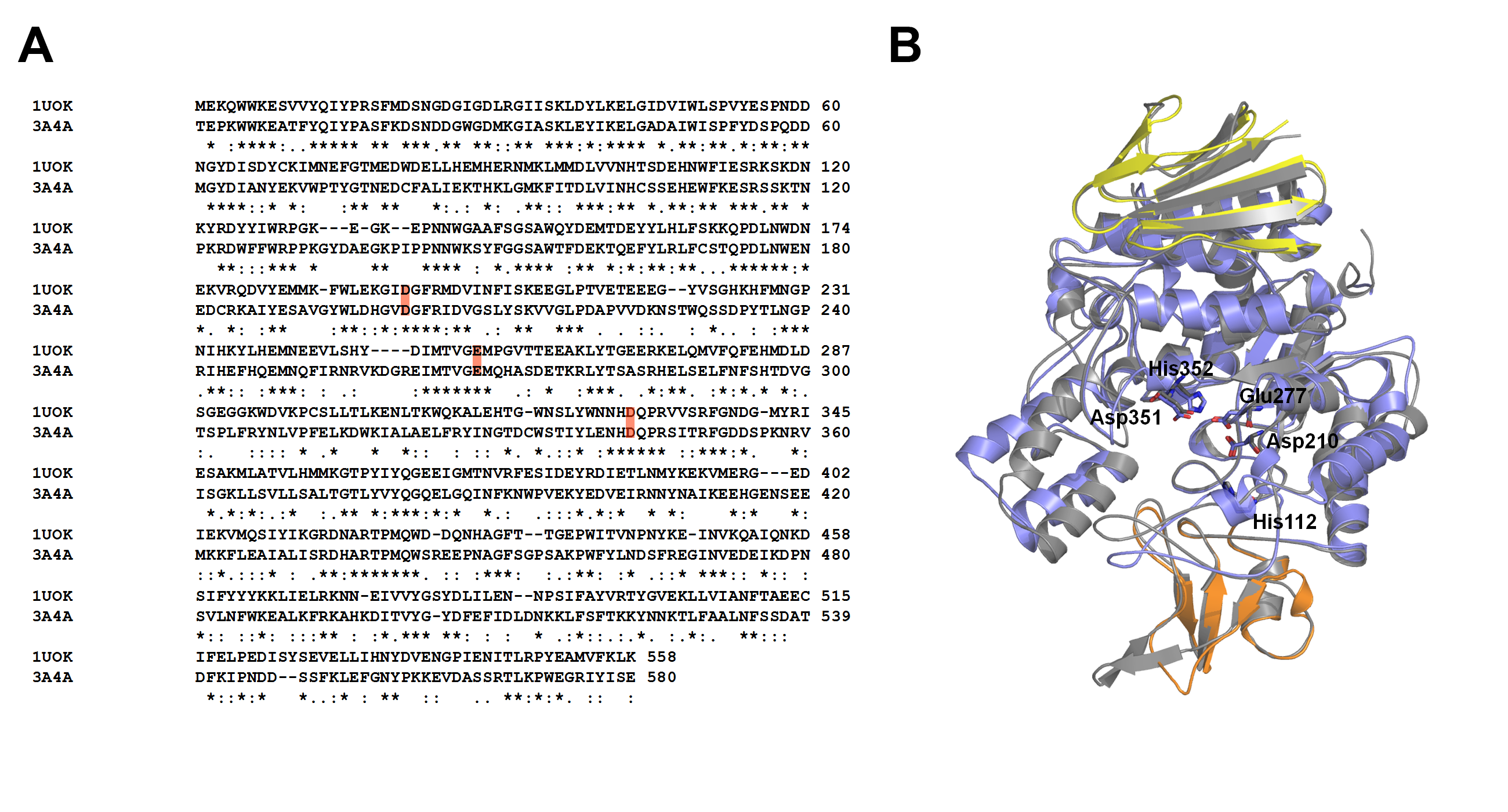

Supplement: Figure S1 — Sequence alignment and homology modeling structure of S. cerevisiae isomaltase using a template B. cereus oligo-1,6-glucosidase. (A) Sequence alignment of S. cerevisiae isomaltase (represented as 3A4A) with oligo-1,6-glucosidase (1UOK). The catalytic residues are indicated in a red box. (B) Comparative view of the homology modeled structure of S. cerevisiae isomaltase constructed by the template with its own crystal structure (PDB ID: 3A4A). The conserved catalytic residues represented as sticks. The N-terminal, subdomain, and C-terminal domains for the homology model are shown in blue, orange, and yellow, respectively. The crystal structure of isomaltase is colored by black. (TIF) [file pone.0085827.s001.tif]

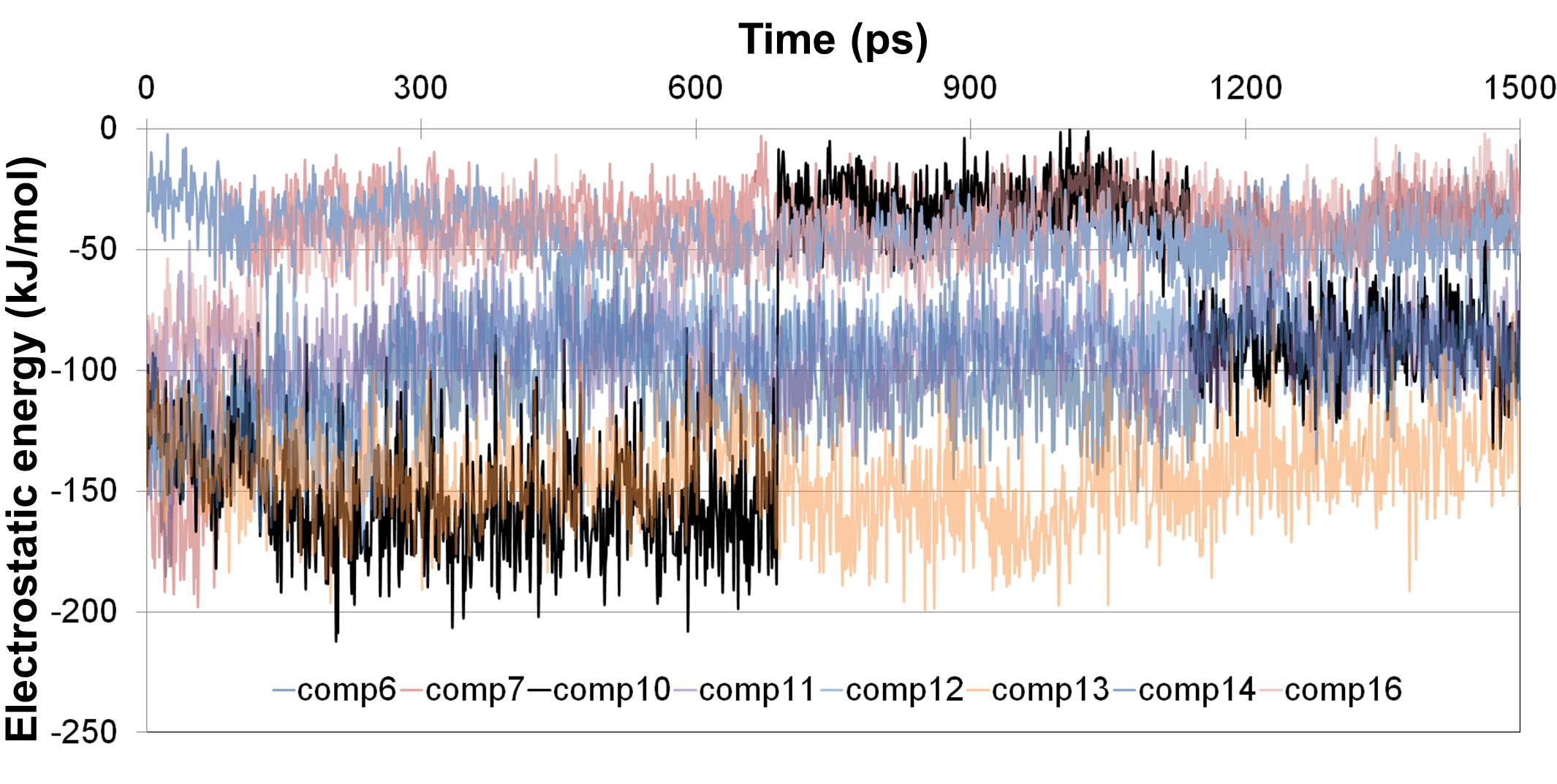

Supplement: Figure S2 — Electrostatic energy plot of all systems during the 1.5 ns simulation time. Energy values for all the other compounds are represented as transparent colors to highlight the energy values for compound 10. (TIF) [file pone.0085827.s002.tif]

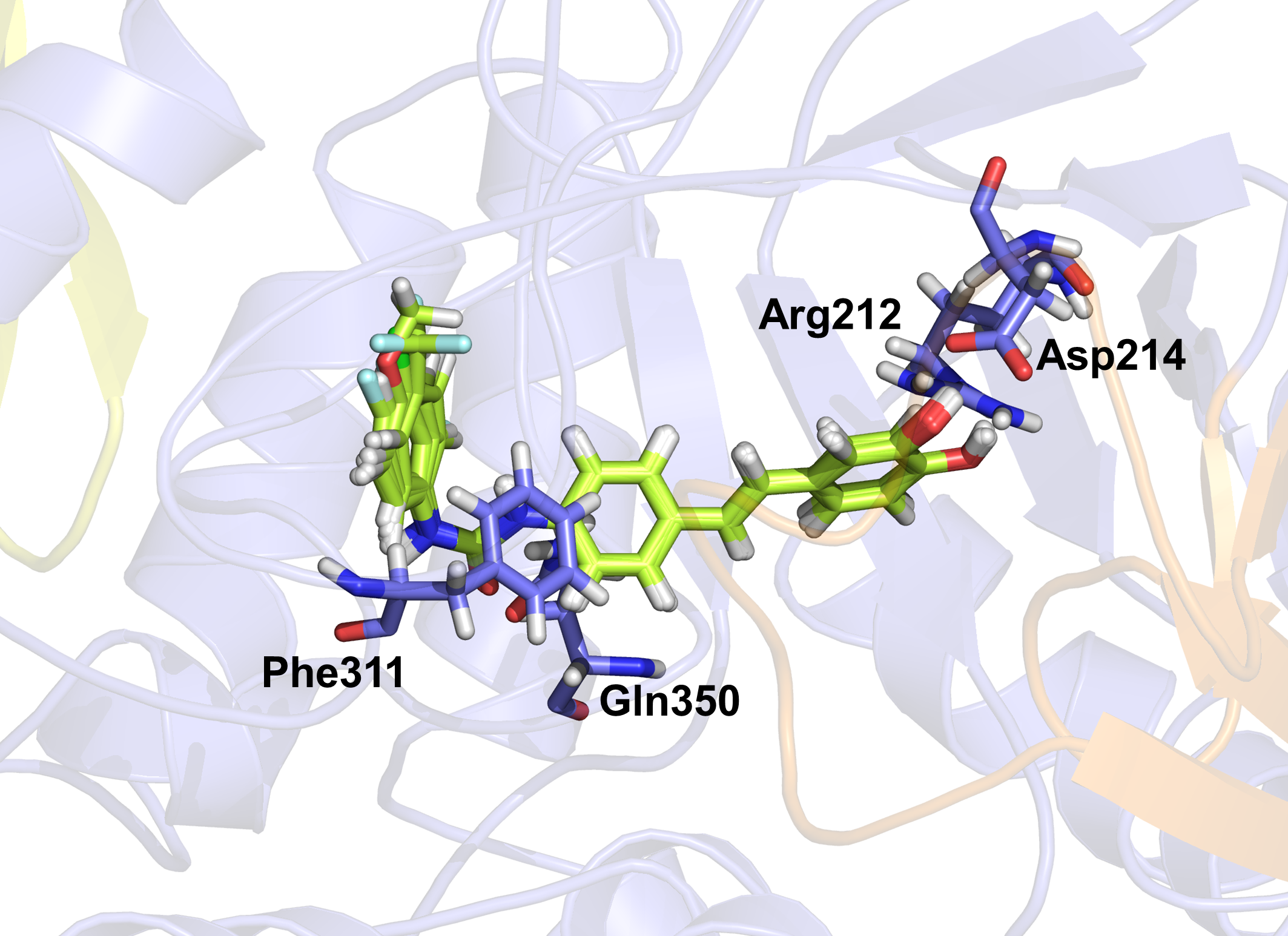

Supplement: Figure S3 — Docking poses of stilbene derivatives in adjusted protein structure of the second trial system, which is the lowest energy conformation, with interacting residues which are highlighted by violet sticks. (TIF) [file pone.0085827.s003.tif]

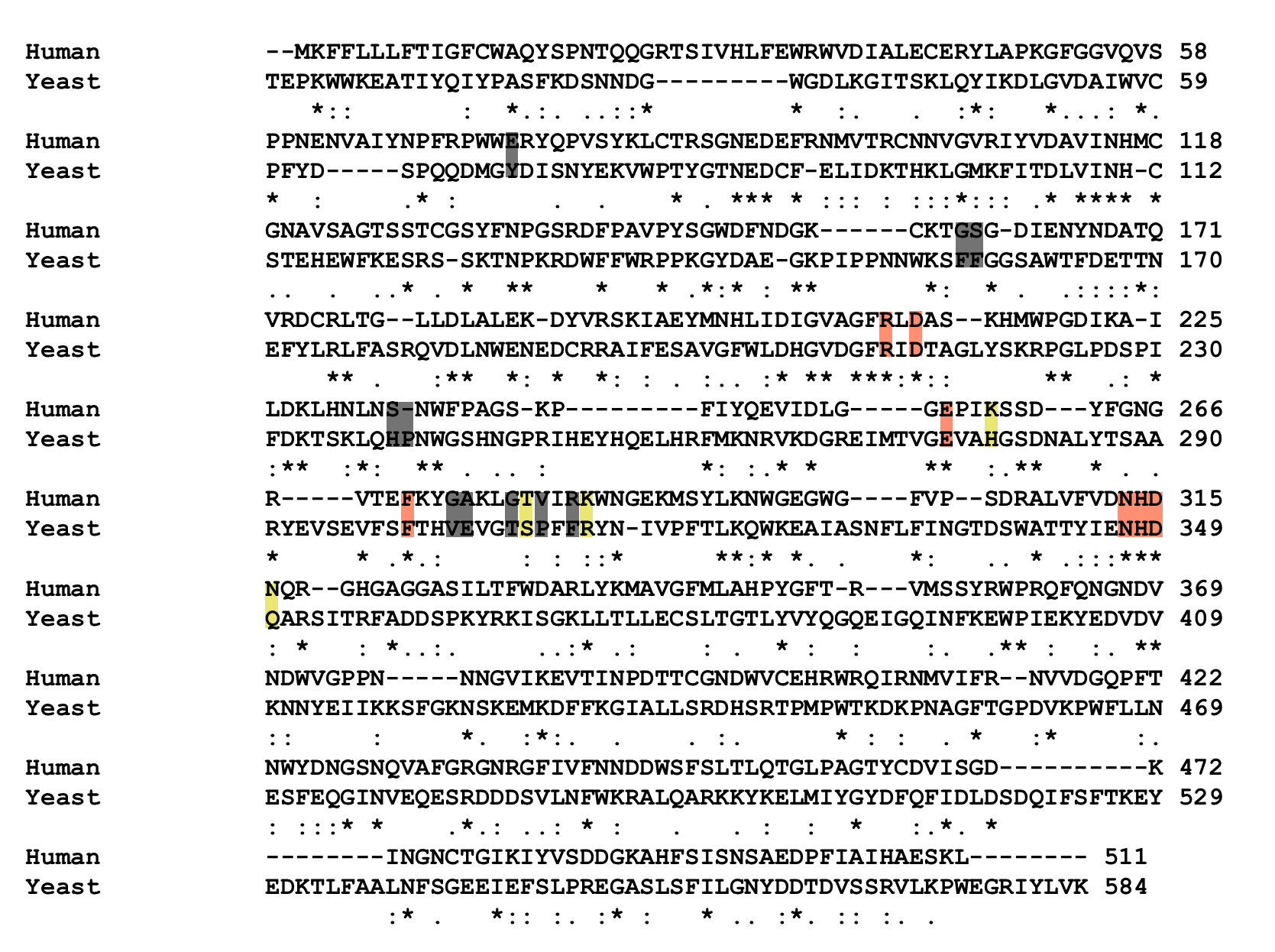

Supplement: Figure S4 — Sequence alignment of S. cerevisiae α-glucosidase (represented as YEAST) with human α-amylase (Human). Each identical, conserved, and non-conserved interacting residue is indicated in a red, yellow, and black box, respectively. Sequence identities are denoted by asterisks (*), conservative substitutions by colons (:), and semi-conservative substitutions by dots (.). (TIF) [file pone.0085827.s004.tif]
