# Supplementary material for: Binding Mode Analyses and Pharmacophore Model Development for Stilbene Derivatives as a Novel and Competitive Class of α-Glucosidase Inhibitors
Source: PLoS One. 2014 Jan 21;9(1):e85827. doi: 10.1371/journal.pone.0085827 (PMC3897524; doi:10.1371/journal.pone.0085827)
Supplement: Table S1 — Interaction energy difference obtained by comparing mostly similar frames excepting π-sigma interaction. (DOCX) [file pone.0085827.s005.docx]

| First frame (ps) | Comp6 E304_CB (nm) | Comp6 E304_CG (nm) | Second frame (ps) | Comp6 E304_CB (nm) | Comp6 E304_CG (nm) | Distance difference (nm) | Energy difference (kJ/mol) |
| --- | --- | --- | --- | --- | --- | --- | --- |
| 9784 | 0.47 | 0.36 | 9785 | 0.56 | 0.54 | 0.27 | -26.69 |
| 1220 | 0.40 | 0.41 | 1219 | 0.53 | 0.54 | 0.25 | -17.02 |
| 5159 | 0.45 | 0.42 | 5160 | 0.57 | 0.55 | 0.24 | -12.30 |
| 6420 | 0.45 | 0.39 | 6419 | 0.55 | 0.52 | 0.23 | -20.07 |
| 1119 | 0.42 | 0.44 | 1118 | 0.56 | 0.54 | 0.23 | -3.81 |
| 1269 | 0.37 | 0.41 | 1270 | 0.51 | 0.50 | 0.23 | -13.58 |
| 345 | 0.42 | 0.45 | 346 | 0.54 | 0.56 | 0.23 | -4.22 |
| 9587 | 0.44 | 0.41 | 9586 | 0.55 | 0.53 | 0.22 | -26.92 |
| 786 | 0.42 | 0.40 | 785 | 0.51 | 0.53 | 0.22 | -35.73 |
| 5137 | 0.47 | 0.49 | 5138 | 0.59 | 0.59 | 0.22 | -10.53 |
| 262 | 0.45 | 0.46 | 261 | 0.54 | 0.59 | 0.22 | -35.68 |
| 631 | 0.43 | 0.43 | 630 | 0.52 | 0.56 | 0.22 | -22.90 |
| 752 | 0.43 | 0.38 | 753 | 0.52 | 0.50 | 0.21 | -5.91 |
| 7971 | 0.44 | 0.42 | 7972 | 0.53 | 0.54 | 0.21 | -6.50 |
| 5237 | 0.46 | 0.48 | 5238 | 0.56 | 0.59 | 0.21 | -2.52 |
| 1976 | 0.41 | 0.42 | 1975 | 0.52 | 0.52 | 0.20 | -5.79 |
| 9776 | 0.46 | 0.45 | 9777 | 0.57 | 0.55 | 0.20 | -8.06 |
| 5438 | 0.43 | 0.45 | 5437 | 0.53 | 0.54 | 0.20 | -1.36 |
| 5118 | 0.47 | 0.46 | 5119 | 0.60 | 0.54 | 0.20 | -1.53 |

**Table S1.** Interaction energy difference obtained by comparing mostly similar frames excepting π-sigma interaction

CB, β-carbon; CG, γ-carbon
